# Assessing the quality, reliability, and transparency of YouTube videos on spiritual palliative care: a content analysis

**DOI:** 10.3389/fpubh.2025.1497855

**Published:** 2025-02-11

**Authors:** Fhaied Almobarak

**Affiliations:** Department of Fundamentals of Nursing, College of Nursing, Imam Abdulrahman Bin Faisal University, Dammam, Saudi Arabia

**Keywords:** quality, reliability, transparency, JAMA, Modified DISCERN, spirituality, palliative care, nursing

## Abstract

**Aim:**

To inspect the quality, reliability, and transparency of YouTube videos on spiritual palliative care by employing systematic scoring benchmarks, such as JAMA and Modified DISCERN.

**Background:**

Spiritual care is vital in palliative care, and YouTube is a popular platform for health information, though the quality of such content remains unexplored. The present study is the first analysis of spiritual palliative care videos on YouTube, revealing the types of creators (e.g., educational institutions, healthcare providers, etc.), dominant video formats (documentaries, testimonials, lectures, etc.), and overall quality and transparency of the content.

**Methods:**

On 19th April 2024, a sample of 50 spiritual palliative care YouTube videos was compiled and examined for quality, reliability, and transparency assessment. JAMA and Modified DISCERN scoring systems were used to score the sample videos. The first step in data analysis was determining the variables’ normality. For this, the Shapiro–Wilk test was employed. The data was not normally distributed, so group-wise comparisons and correlation analysis were conducted using non-parametric methods. Correlation analysis was done using the Spearman’s test. The quantitative data of the groups/categories were compared using the Kruskal-Wallis test.

**Results:**

The current study’s findings showcase that the quality, reliability, and transparency of the spiritual palliative care YouTube videos are moderate. The mean JAMA score for transparency was slightly better than the mean Modified DISCERN score for the quality/reliability of the sample videos.

**Impact:**

The current study addresses the ambiguity about the quality, reliability, and transparency of spiritual palliative care videos on YouTube. The results highlight the lack of superior quality, reliability, and transparency of the available video content related to the subject. Concerned authorities must recognize the risk of contact with substandard quality health-related YouTube content. To promote public health, initiatives are needed to increase patients’ chances of access to high-quality YouTube information about the spiritual facet of palliative care.

## 1 Introduction

Palliative care is a multifaceted approach to specialized medical and nursing care for people with life-threatening illnesses. Its main emphasis is on easing the symptoms and stress of the ailment while also upgrading the quality of life for the patient and the family. Palliative care encompasses a holistic approach focusing on a broad spectrum of patient needs: physical, emotional, social, psychological, and spiritual (1). Recent research delineates that palliative care’s focal point is improving patients’ overall quality of life while implementing curative procedures associated with their life-threatening illnesses. Thus, it offers a support system to the patients, including relief from pain and other distressing symptoms, while incorporating psychological and spiritual facets of patient care (2).

There is no denying the significance of spiritual care as a vital component of palliative care practice, as it helps to provide a sense of peace, meaning, and connection to the transcendent or something superior to oneself. The spiritual dimension is often overlooked even though it is a crucial aspect with extraordinary benefits for patients and caregivers (3). Spirituality is much more than religious beliefs, comprising personal discovery, self-reflection, and connectedness (4). Spirituality is an inevitable component of high-quality palliative care, as it heals the deep wounds of the patients’ souls by helping them find meaning and purpose in life (5). For many, it is a fount of strength and resilience in enduring the struggles posed by their health conditions (6). Moreover, effective therapeutic success can be achieved by integrating the psychological and spiritual dimensions in adult palliative care (7). The importance of the spiritual dimension concerning nursing practice can be determined by the fact that the previous research considers it on par with physical, social, and psychological domains (8).

In today’s digital era, patients, families, and healthcare providers rely on online mediums for medical-related information. YouTube, a video-sharing platform, is one of the most popular online mediums that offers a plethora of unregulated health-related content. A broad spectrum of content ranging from professional healthcare advice to personal experiences related to various medical conditions, including spiritual palliative care, can be accessed through YouTube. Being expansive in reach and readily accessible, YouTube is an ideal repository for distributing and exploring the nuances of spirituality within the palliative care context.

The variable quality of content available on YouTube raises concerns about the accuracy and reliability of the information disseminated (1, 9, 10). The unregulated nature of YouTube videos is a concern that needs to be addressed. Therefore, it is pertinent to systematically evaluate the quality and reliability of the content to ensure that users have access to accurate and comprehensive information. For evaluation, it is essential to employ well-recognized and established quality assessment tools like GQS, the Journal of the American Medical Association (JAMA), and Modified DISCERN scoring systems to analyze online content on various health topics (1, 11–16). These scoring systems are structured approaches designed to fathom the transparency, reliability, and quality of health information in online videos, considering various factors such as the accuracy of content, the credibility of sources, and the comprehensiveness of the information delivered.

Keeping in mind the unsurmountable evidence that points toward the importance of spiritual palliative care and the mounting reliance on digital media for medical information, this research intends to evaluate the quality/reliability and transparency of YouTube videos on spiritual palliative care by applying the standardized JAMA and Modified DISCERN scoring systems. This study sets out to fill the gap in the existing literature by systematically analyzing YouTube content related to the spiritual facet of palliative care. No research has been done to assess the quality and reliability of YouTube videos that give patients seeking spiritual palliative care relevant medical information. Evaluating the caliber of pertinent videos about spiritual palliative care is essential to figuring out whether YouTube videos are potentially helpful.

In conclusion, the proposed research will aid in understanding the quality, reliability, and transparency of spiritual palliative care resources available on YouTube. By using established quality assessment tools, this study will offer insights into the existing state of video content in this area and educate future endeavors to upgrade the transparency, reliability, and quality of information for those seeking guidance on spiritual aspects of palliative care. This assessment will facilitate the identification of top-notch spiritual palliative care resources and potentially guide the patients and healthcare providers toward it. It will also help to detect areas where additional, reliable, and more informative content is needed.

## 2 Background

Spirituality has long been interweaved with palliative care, targeting to address the holistic needs of patients facing life-threatening illnesses. Previous studies have shed light on the significance of spirituality in palliative care. The existing literature emphasizes the eminence of its role in addressing patients’ and families’ existential requirements. Researchers suggest that patients and their relatives can achieve positive outcomes by integrating spiritual touch into palliative services (17, 18). Moreover, there is mounting recognition of the influence of religion/faith on subduing terminal illness-related emotional challenges (such as anxiety and fear) (19). To cater to diverse religious and cultural backgrounds, the Diamond Model has been introduced to offer a non-religious approach to meet patients’ spiritual needs (20). In short, existing research elevates the importance of spiritual and religious guidance in palliative care, stressing the need for more rigorous research to improve interventions and training strategies (21).

Several challenges are posed while integrating spirituality into palliative care. A significant challenge is the lack of systematic and scientific non-religious approaches to address patients’ existential needs (20). Many other barriers hinder the provision of spiritual care. These barriers include late referral for palliative care, work overload, uncontrolled physical symptoms, etc. (10). Addressing these challenges could lead to improved patient outcomes and more inclusive delivery of spiritual palliative care (17, 22–24). Specific strategies can be chalked out to overcome obstacles in spiritual care provision. More thorough research on different patient groups and the importance of training healthcare providers about various religions and cultures are underlined as vital strategies (18, 19). The palliative care teams should be comprehensively briefed about the definitions, guidelines, and evidence related to the role of spiritual and religious factors in serious illness (25). Fostering seamless spiritual care delivery within palliative care comes with both opportunities and challenges.

Research into the quality and transparency of information related to spiritual palliative care on YouTube is warranted due to the platform’s extensive use as a source of health information (1, 26, 27). The importance of spiritual needs in palliative care has been substantiated in recent research (3, 17, 28). However, it is essential in this digital era to gauge whether the content available on YouTube is reliable and valuable for patients, families, and healthcare providers. Unfortunately, while YouTube is a popular informational resource, studies have revealed that the quality and accuracy of medical/health information on the platform can be compromised (26, 27, 29). Moreover, the evidence suggests that healthcare professionals’ higher quality, reliable, and accurate videos tend to get lower viewership than those uploaded by non-medical sources (26, 27). This inconsistency underscores the need for research to pinpoint and promote higher-quality, reliable spiritual palliative care content on YouTube. In short, there is a dire need to investigate the quality, reliability, and transparency of spiritual palliative care information on YouTube to ensure viewers can access reliable and comprehensive materials. Such research could lead to developing content creation and curation guidelines, ultimately improving the support available to those seeking spiritual care in palliative care.

## 3 The study

### 3.1 Aims

This study examines spiritual palliative care YouTube videos’ quality/reliability and transparency by employing systematic scoring benchmarks, such as JAMA and Modified DISCERN. The secondary objective of this research was to distinguish the quality/reliability and transparency of spiritual palliative care YouTube videos according to the upload source and video type.

### 3.2 Design

The current research design is based on qualitative content analysis of spiritual palliative care YouTube videos. This study uses JAMA and Modified DISCERN scoring systems to assess the transparency and reliability of spiritual palliative care videos on YouTube. Previous researchers have used Modified DISCERN and JAMA instruments to analyze the reliability and transparency of medical-related YouTube videos. The original DISCERN system consists of 15 questions. A modified version derived from the original was introduced in 2012 (30). The modified version consists of 5 questions that help gauge the quality and reliability of the information the online videos provide. The questionnaire consists of the following items: 1. Are the aims clear and achieved? 2. Are reliable sources of information used (i.e., publication cited)? 3. Is the information presented balanced and unbiased? 4. Are additional sources of information listed for patient reference? 5. Are areas of uncertainty mentioned? Each of the five questions gets assigned one score if the answer is “yes” or a zero score for a “no” answer. So, each video was scored between 0 and 5 in total.

The JAMA benchmark assesses the quality and transparency of online information using four distinct measures, including authorship (authors, contributors, affiliations, and credentials), attribution (references and sources used for the content and copyright information), disclosures (sponsorship, advertising, commercial funding, and potential conflicts of interest), and currency (dates of upload and updated information). One point is awarded for each of the criteria met. The resulting scores range between 0 and 4. A higher total score indicates better quality for both scoring systems and vice versa.

### 3.3 Sample

The sample used for the present research analysis comprised 50 (*N* = 50) YouTube videos related to spiritual palliative care. All the videos were closely analyzed and scored according to the Modified DISCERN and JAMA criteria. Moreover, the hyperlink, title, count of views, length in seconds, count of likes, count of comments, source of upload, and video type were recorded for each video.

### 3.4 Data collection

**Figure 1:**
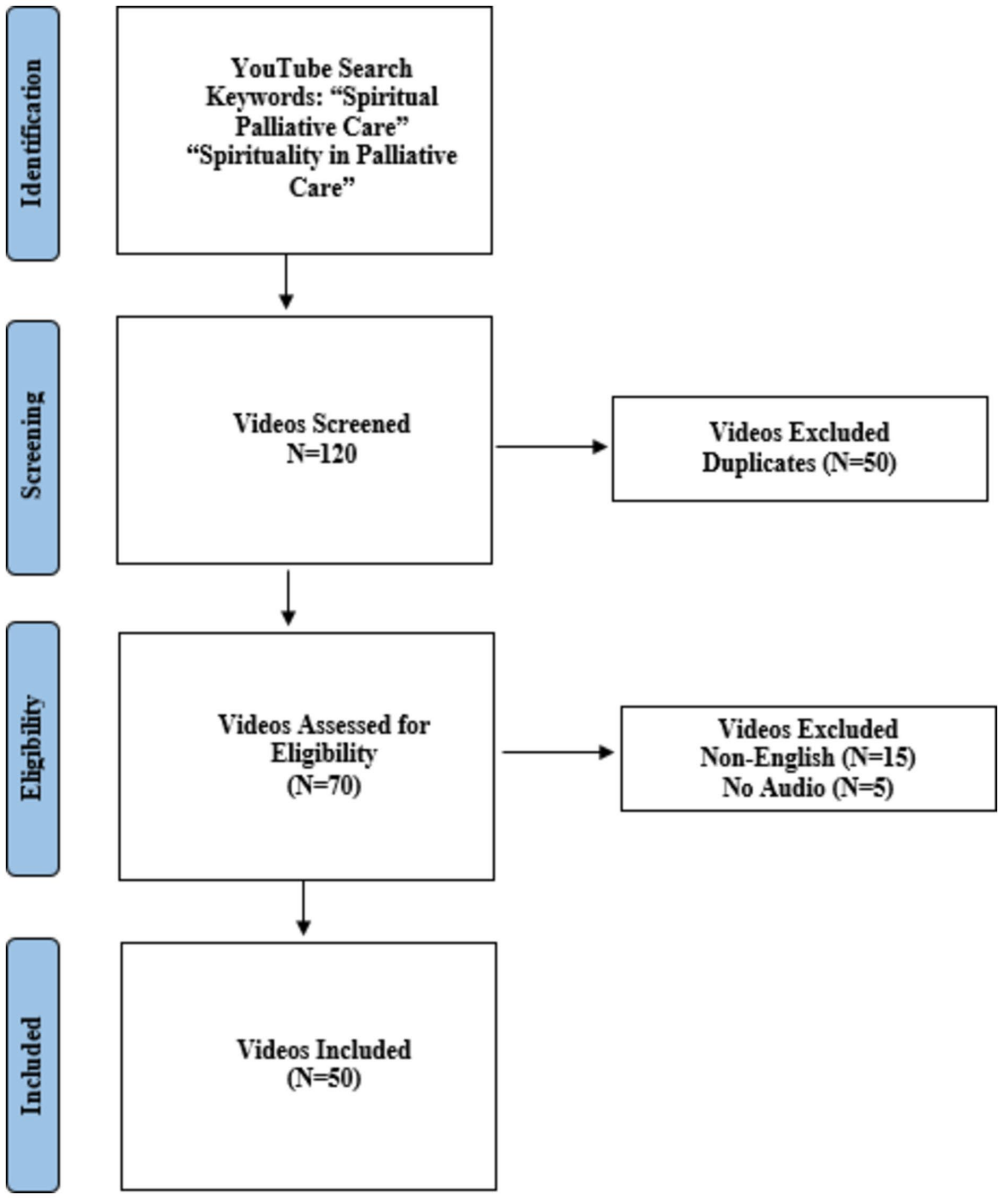
Research flowchart of the videos included.

On 19th April 2024, a video-based search on the YouTube online hosting platform^1^ was performed by using the English keywords: “spiritual palliative care” and “spirituality in palliative care.” Search history and cache were cleared before initiating the search, and the search was performed in incognito mode. Videos were sorted based on relevance. Videos unrelated to spiritual palliative care and in languages other than English were excluded from the evaluation. The videos without audio were also removed from the sample. The first 60 eligible videos for each keyword were selected. From the 120 videos (60 from each keyword search), duplicates (*N* = 50), videos without audio (*N* = 5), and non-English videos (*N* = 15) were removed. Hence, the final sample size was *N* = 50, which was evaluated using methods from previous studies. The research flowchart is presented in Figure 1.

After evaluating the videos for eligibility, the sample videos were shared with two trained professionals specializing in spiritual palliative care for scoring. Both qualified reviewers viewed and scored the videos independently. Therefore, the inter-rater reliability was calculated using Cohen’s Kappa coefficient to determine the level of agreement among raters. The range of the coefficient varies between zero and one. The nearer the value to 1.0, the better the interrater reliability. The Cohen’s Kappa coefficient for reliability between the raters was 0.87 and 0.89 for JAMA and Modified DISCERN scores, respectively.

### 3.5 Data/statistical analysis

The Statistical Package for Social Sciences (SPSS) 29.0 (IBM Corp., Armonk, NY) was used for data analysis. The descriptive statistics enlist the characteristics of the videos, such as the count of views, count of likes, length in seconds, count of comments, source of upload, and video type. The results for descriptives were reported as frequency, percentage, mean, standard deviation, median, etc. The upload source classified the uploaders’ profiles under four categories: healthcare organization, religious channel, educational institution, and news agency. The video type distinguished the content of the videos into three categories: educational, documentary, and testimonial. Data analysis commenced by assessing the sample variables’ normality. Shapiro–Wilk test was used for this purpose. The results of normality analysis revealed that the data was not normally distributed. Therefore, non-parametric techniques were employed for correlation and group-wise comparison analyses. The Spearman’s test was conducted for correlation analysis. The Kruskal–Wallis test was used to compare quantitative data of the groups/categories. The level of statistical significance was set at *p* < 0.05, and findings were also interpreted as *p* < 0.01 and *p* < 0.001 when applicable.

### 3.6 Ethical considerations

Using publicly available video clips as research data and reporting quality analysis results does not require ethical committee approval. Accordingly, the study was not subjected to ethical review.

### 3.7 Rigor

The SRQR EQUATOR network guidelines were adhered to ensure the research’s rigor and transparency in reporting.

## 4 Findings

The characteristics of the sample videos are presented in Table 1. These descriptive statistics give a fleeting overview of the mean, median, standard deviation, range, minimum, and maximum values of the Modified DISCERN score, JAMA score, count of views, count of likes, count of comments, and length (seconds). It is crucial to note that the mean Modified DISCERN and JAMA scores are 2.76 (SD = 1.45) and 2.86 (SD = 0.55), respectively. These mean scores indicate intermediate overall quality, reliability, and transparency of spiritual palliative care YouTube videos.

**Table 1:** Characteristics of sample videos (*N* = 50).

	Mean	Median	SD	Range	Min	Max
DISCERN	2.76	3.00	1.45	5.00	0.00	5.00
JAMA	2.86	3.00	0.55	2.00	2.00	4.00
Count of views	2497.38	923.50	4784.17	30592.0	8.00	30600.00
Count of likes	23.06	9.50	45.64	285.00	0.00	285.00
Count of comments	0.82	0.00	2.69	14.00	0.00	14.00
Length (seconds)	1675.74	661.00	1,875.30	8989.00	30.00	9019.00

**Figure 2:**
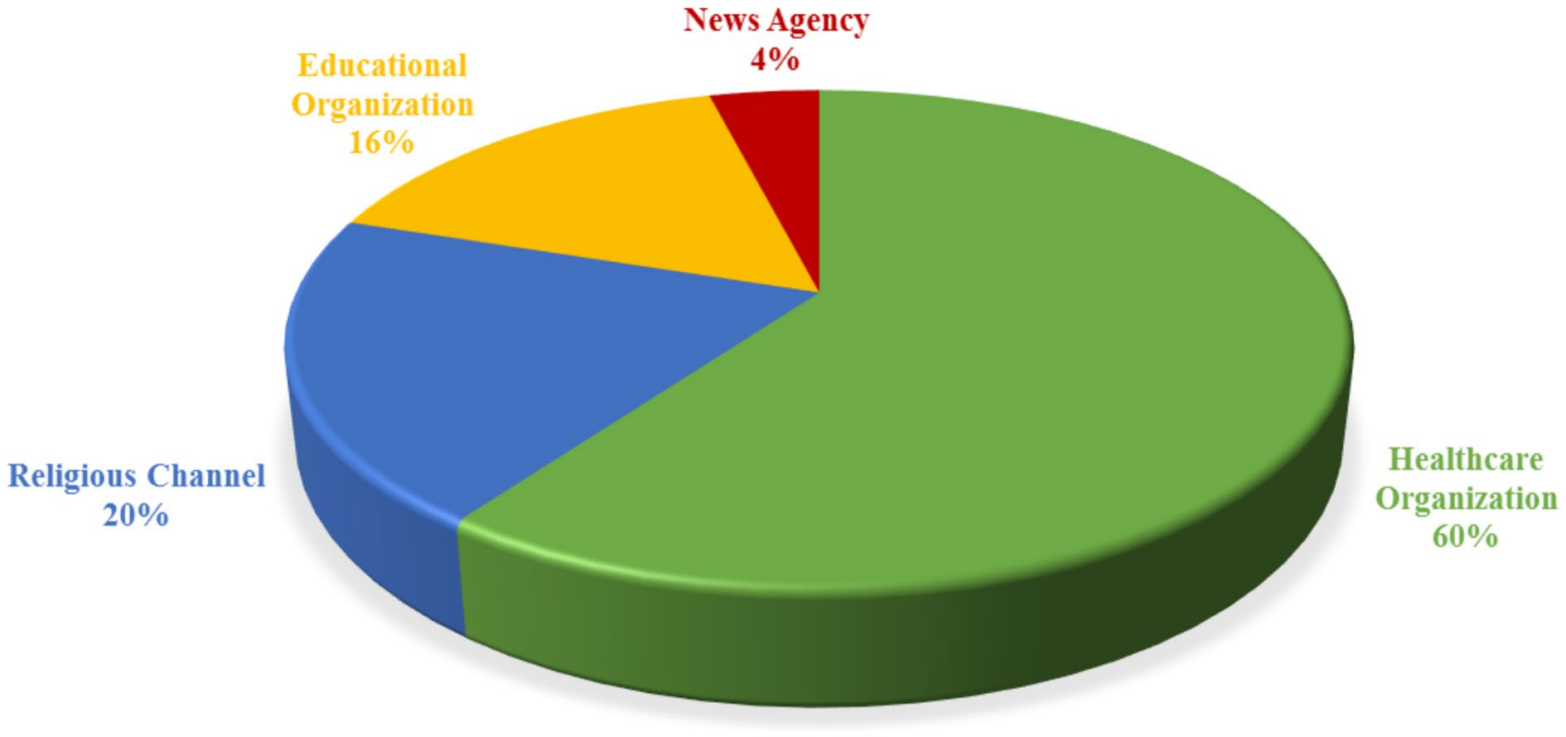
Distribution of videos according to upload source.

Figure 2 depicts that healthcare organizations uploaded 60% of the spiritual palliative care videos, followed by religious channels (20%), educational organizations (16%), and news agencies (4%).

**Figure 3:**
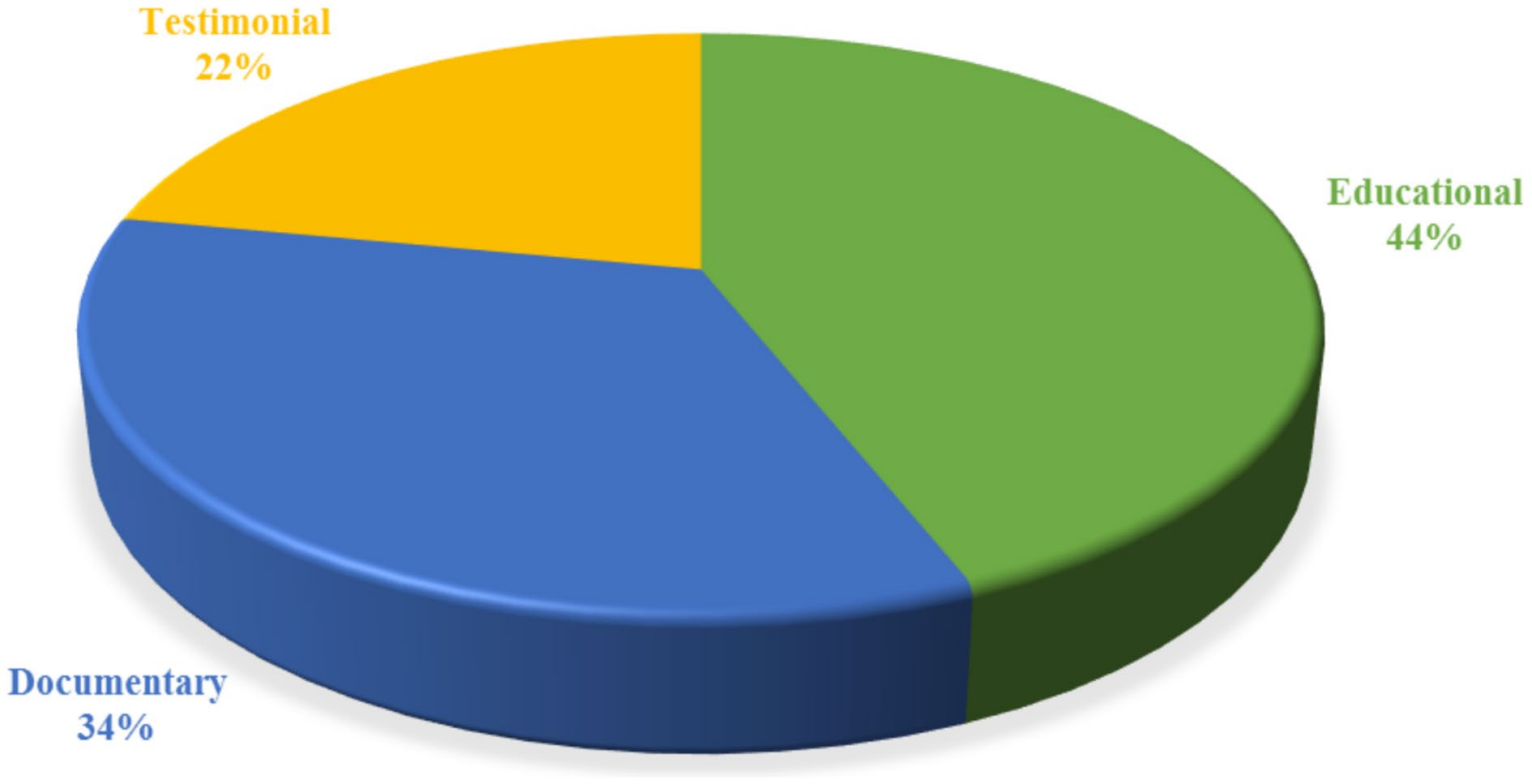
Distribution of videos according to video type.

According to the stats shown in Figure 3, 44% of the content was educational. Documentary and testimonial content was 34 and 22%, respectively.

Table 2 enlists Spearman’s correlation coefficients. A single or double asterisk symbol indicates the significance level of correlation. The Modified DISCERN score significantly correlates with the JAMA score and the length of the videos. There is also a significant correlation between the JAMA score and the length of videos. A significant correlation also exists between the count of likes, the count of comments, and the count of views.

**Table 2:** Spearman’s pairwise correlation matrix.

		JAMA	DISCERN	Count of views	Count of likes	Count of comments	Length
JAMA		1					
DISCERN		0.372**	1				
Count of views		−0.046	0.086	1			
Count of likes		−0.007	0.069	0.827**			
Count of comments		−0.014	0.170	0.359*	0.415**	1	
Length		0.305*	0.799**	0.069	0.126	0.128	1

**Correlation is significant at the 0.01 level (2-tailed). *Correlation is significant at the 0.05 level (2-tailed).

**Figure 4:**
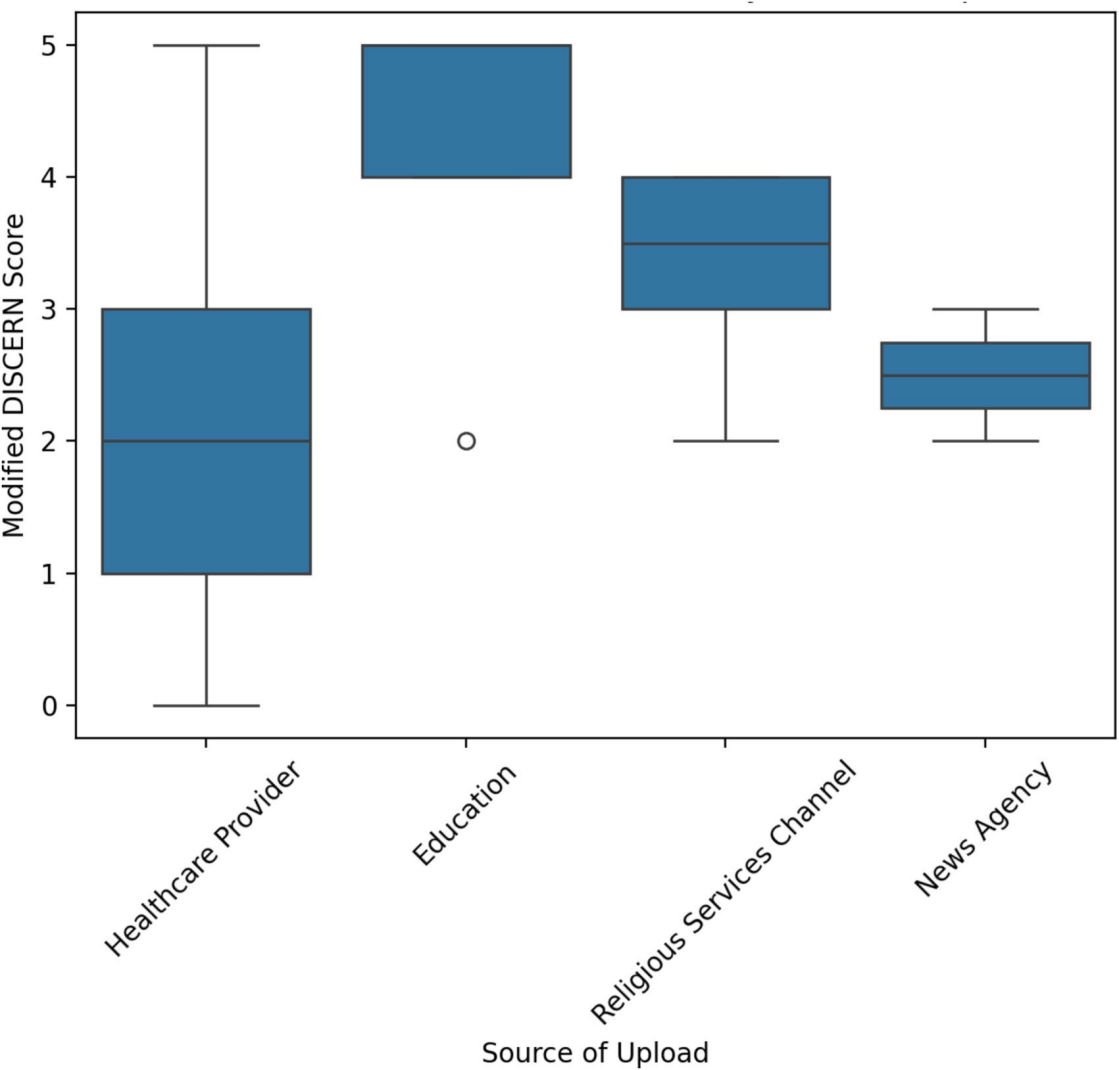
Box and Whisker plot for Kruskal-Wallis H ranks according to upload source.

**Figure 5:**
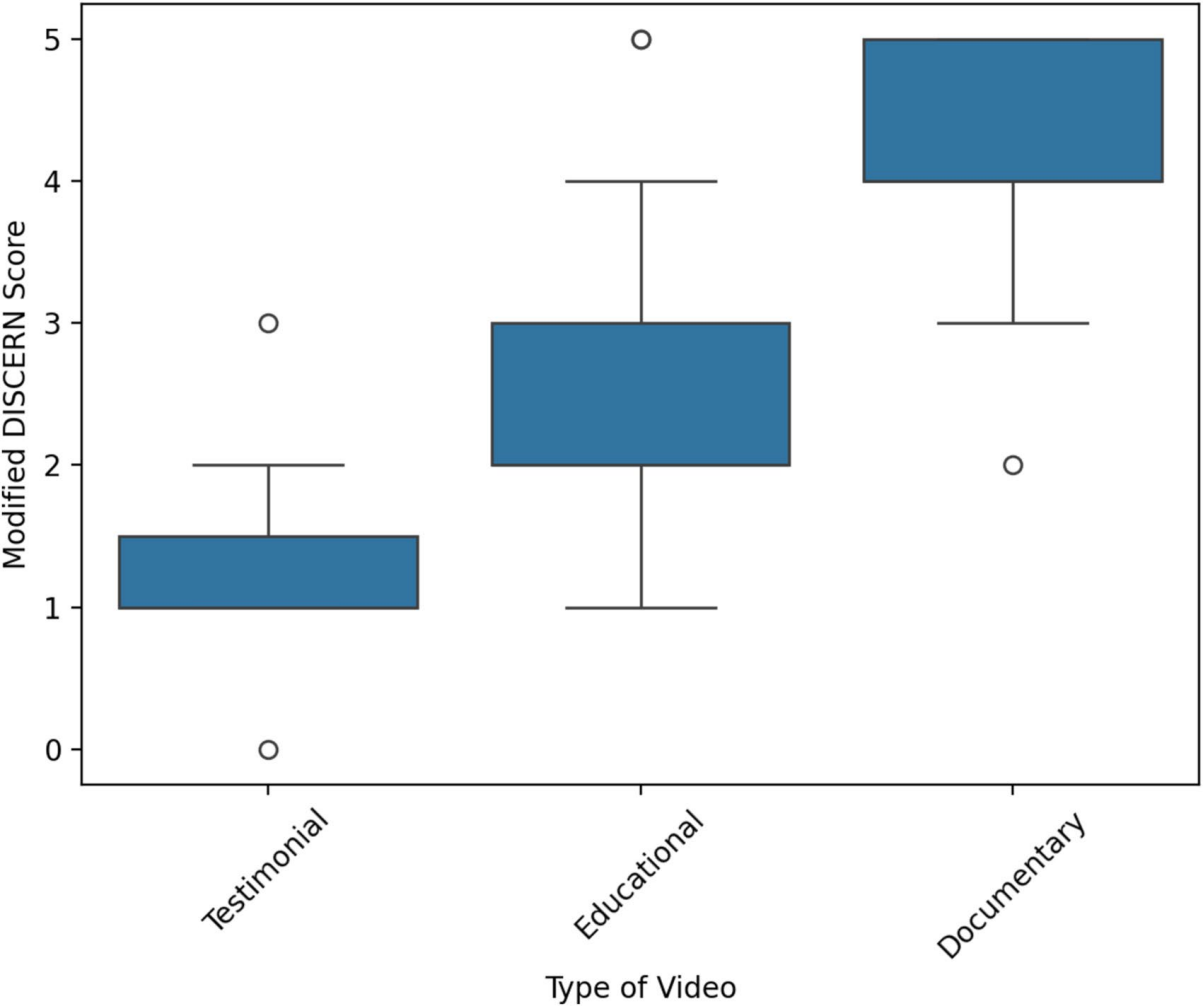
Box and Whisker plot for Kruskal-Wallis H ranks according to video type.

Kruskal-Wallis H test showed a statistically significant difference in Modified DISCERN score between the different categories of upload source and video type. The results imply that the reliability of spiritual palliative care videos uploaded by educational organizations was the highest according to the Modified DISCERN criteria, followed by religious channels, news agencies, and healthcare organizations. Moreover, the highest quality of the spiritual palliative care videos was documentary, followed by educational and testimonial content. In contrast, a statistically insignificant difference in JAMA score between the upload sources and video type was reported. Kruskal-Wallis H test results are presented in Tables 3, 4 and graphically in Figures 4, 5.

**Table 3:** Kruskal-Wallis H ranks according to upload source.

	Source of upload	N	Mean rank	Kruskal-Wallis H
JAMA	Healthcare organization	30	24.93	0.738
	Religious channel	10	24.40	
	Educational organization	8	28.13	
	News agency	2	29.00	
	Total	50		
DISCERN	Healthcare organization	30	19.60	16.368*
	Religious channel	10	31.10	
	Educational organization	8	41.19	
	News agency	2	23.25	
	Total	50		

*Asymptotic significance level < 0.001.

**Table 4:** Kruskal-Wallis H ranks according to video type.

	Video type	N	Mean rank	Kruskal-Wallis H
JAMA	Educational	22	22.57	5.860
	Documentary	17	31.09	
	Testimonial	11	22.73	
	Total	50		
DISCERN	Educational	22	22.50	27.675*
	Documentary	17	38.91	
	Testimonial	11	10.77	
	Total	50		

*Asymptotic significance level < 0.001.

## 5 Discussion

The results and findings of the current study showcase that the reliability and transparency of the spiritual palliative care YouTube videos are average. The mean JAMA score was slightly better than the mean Modified DISCERN score of the sample videos. The educational organizations uploaded the highest-quality videos, while the religious channels, news agencies, and healthcare organizations lagged behind. Documentarian content ranked higher for the Modified DISCERN score, followed by educational and testimonial content.

The findings of the present research are in line with the outcomes reported by some recent works. The latest empirical research indicates the existence of average or below-average-quality medical and health-related videos on YouTube. Studies assessing videos related to different medical-related topics, such as postpartum pain management, tumors, TOETVA, etc., reported low quality and accuracy, with content often coming from unknown resources (26, 27, 31, 32).

YouTube can be a resourceful tool for educating and informing people about health-related topics, including the spiritual facet of palliative care. However, the present study findings suggest otherwise. In short, the current research has some fruitful implications. For instance, there is an undeniable need to develop content creation guidelines, standardize the information available, and ultimately optimize the quality, reliability, and transparency of spiritual palliative care-related YouTube content. Policymakers are responsible for promoting public health by addressing misinformation and improving access to quality healthcare information. This study highlights the need for policies or guidelines that encourage creating and disseminating reliable spiritual palliative care content on platforms like YouTube. Policymakers can collaborate with healthcare professionals and content creators to develop standards for information accuracy, transparency, and ethical considerations in health-related videos. This collaboration can help bridge the gap between online health information and evidence-based practices, benefiting patients and caregivers. Healthcare professionals should also be vigilant in acknowledging and discussing the risk of contact with substandard quality and unreliable YouTube-based spiritual care information when consulting patients who seek palliative care. Healthcare providers play a crucial role in ensuring that patients receive accurate and reliable information, especially when it comes to sensitive topics like spiritual care in palliative settings.

### 5.1 Limitations

Like any other research, the current study also has a few demerits. Firstly, non-English videos were excluded from the sample. Secondly, the sample size is relatively small. Thirdly, only two reviewers viewed and scored the videos. Fourthly, merely two phrases, “spirituality in palliative care” and “spiritual palliative care,” were used for the search strategy, which may or may not represent the searches general people make on this topic. Lastly, the search was conducted at a single point in time, that is, 19th April 2024. Future research on spiritual palliative care can opt for a cross-sectional analysis of YouTube videos through searches at multiple time points and address the other limitations mentioned above.

## 6 Conclusion

The present study is the first to give insights into the quality, reliability, and transparency of the spiritual facet of palliative care YouTube content. Most of the YouTube videos discussing spiritual palliative care are of low to moderate quality, so patients should be cautious about trusting the information available on YouTube. Digital literacy training would be advantageous for lay audiences in identifying trustworthy information. Content creators should be encouraged to provide clear and accurate information about spiritual palliative care, including reliable sources and references. Fostering collaborations between healthcare professionals and digital media experts can help to ensure that videos meet medical standards while remaining accessible and engaging.

The findings of the current study suggest that the videos uploaded by educational organizations were superior in quality compared to religious channels, healthcare organizations, and news agencies. Thus, the videos uploaded by educational organizations should be preferentially recommended to patients seeking online information related to spiritual palliative care. Moreover, inclusive, regulated, and informative videos covering the spiritual facet of palliative care should be promoted.

The current study’s findings can guide healthcare providers to assess and recommend high-quality resources to their patients. They can use the identified criteria and scoring systems to evaluate the reliability, accuracy, and comprehensiveness of spiritual palliative care information on YouTube. By doing so, they can empower patients and their families to make informed decisions about their care and support.

## Data Availability

The original contributions presented in the study are included in the article/supplementary material, further inquiries can be directed to the corresponding author/s.

## Footnotes

^1^ https://www.youtube.com/
